# Member announcement

**DOI:** 10.3109/03009734.2010.505772

**Published:** 2010-07-19

**Authors:** Torbjörn Karlsson, Anna Rask-Andersen

Dear Members,

On Wednesday September 22, the Uppsala Medical Society will arrange its traditional annual meeting, as always held close to the birthday of our founder Israel Hwasser (born September 19, 1790). This year the meeting will be featuring the honorary speaker, professor Lars Wiklund, who will give us some experiences from his research on resuscitation. The lecture will be followed by the celebration dinner. A couple of weeks later on Friday October 15, it is time for the Rudbeck seminar. The theme this year will be medical ethics and life style. Professor Per Westermark has been awarded The Rudbeck price this year and he will present a lecture on amyloid research. The winner of the Fernstrom prize for younger researcher, professor Per-Ola Carlsson, will also present a lecture. His field of research has been that of islet transplantation.

We would also take the opportunity to announce that our new home page now has been launched. You find it under the address – www.upsalalakareforening.se. As before you will find details of different events in which our society is engaged, “veckoprogrammet”, on how to become a member and a direct link to our journal. If you have further suggestions on how to improve the web site, please contact us.

